# On homology modeling of the M_2_ muscarinic acetylcholine receptor subtype

**DOI:** 10.1007/s10822-013-9660-8

**Published:** 2013-06-28

**Authors:** Jan Jakubík, Alena Randáková, Vladimír Doležal

**Affiliations:** Department of Neurochemistry, Institute of Physiology v.v.i., Academy of Sciences of the Czech Republic, Prague, 142 00 Czech Republic

**Keywords:** Muscarinic acetylcholine receptor, G-protein coupled receptor, Homology modeling, Binding energy estimation, MM-GBSA

## Abstract

Twelve homology models of the human M_2_ muscarinic receptor using different sets of templates have been designed using the Prime program or the modeller program and compared to crystallographic structure (PDB:3UON). The best models were obtained using single template of the closest published structure, the M_3_ muscarinic receptor (PDB:4DAJ). Adding more (structurally distant) templates led to worse models. Data document a key role of the template in homology modeling. The models differ substantially. The quality checks built into the programs do not correlate with the RMSDs to the crystallographic structure and cannot be used to select the best model. Re-docking of the antagonists present in crystallographic structure and relative binding energy estimation by calculating MM/GBSA in Prime and the binding energy function in YASARA suggested it could be possible to evaluate the quality of the orthosteric binding site based on the prediction of relative binding energies. Although estimation of relative binding energies distinguishes between relatively good and bad models it does not indicate the best one. On the other hand, visual inspection of the models for known features and knowledge-based analysis of the intramolecular interactions allows an experimenter to select overall best models manually.

## Introduction

Muscarinic acetylcholine receptors are present throughout the body, particularly in the central and peripheral nervous systems, and on innervated tissues, e.g. smooth muscles, salivary glands, etc. Muscarinic receptors are involved in a number of physiological processes, and muscarinic transmission malfunctions are manifested as a wide array of pathological conditions. Muscarinic receptors are therefore target for pharmacological intervention for disorders and diseases ranging from vegetative dysfunctions to complex neurological and psychiatric disorders, such as schizophrenia and Alzheimer’s disease [1].

Over the last two decades, intensive research in the field of muscarinic receptors has resulted in the discovery of new compounds that interact with muscarinic receptors in a novel manner [2]. Several of them exhibit unusual behaviors that do not mimic known orthosteric competitive agonists and antagonists. For example, the agonist xanomeline binds to muscarinic receptors in a wash-resistant manner and influences the receptor orthosteric binding site allosterically [3]. The behavior of these compounds is hard to elucidate without an appropriate molecular model. Like other membrane proteins, muscarinic acetylcholine receptors are difficult to crystallize due to low expression levels and difficulties in the crystallization process itself [4]. The crystallographic structure of muscarinic receptors was not available until recently [5, 6]. Several homology models of muscarinic receptors based on the crystal structure of rhodopsin [7, 8] expressed naturally in high levels have been published [9–13]. With the newly available templates of class A of the G-protein-coupled receptors [8, 14–16] it has become possible to design more reliable homology models. In recent years we have developed several homology models of the M_2_ muscarinic receptor based on these templates, using either the Prime program [17] or the YASARA program [18]. An inherent problem of homology models is the way in which their quality is evaluated. The application of internal checks and scores does not enable the experimenter to decide which of the models is better; the only way is to compare model predictions with experimental results. In this study we present 12 homology models of the M_2_ muscarinic receptor, demonstrate a crucial role of the templates and show insufficiency of the available tests to evaluate model accuracy.

## Results

### Homology modeling of muscarinic acetylcholine receptors

Like other membrane proteins, muscarinic acetylcholine receptors are difficult to crystallize due to low expression levels and difficulties in the crystallization process itself [4], and until recently the crystallographic structure of muscarinic receptors was not available [5]. The only G-protein coupled receptors (GPCR) that is naturally expressed in high levels is rhodopsin. Several homology models of muscarinic receptors based on the high-resolution crystal structure of rhodopsin [7, 8] were designed to help explain particular experimental results [9–13]. Thanks to the continuous growth of computing power and improvements in available molecular modeling software, new homology models can now be generated quickly. In recent years, we have designed 12 homology models of M_2_ muscarinic acetylcholine receptors (ver01–ver12) that we present in this study.

### Building the models

**Fig. 1:**
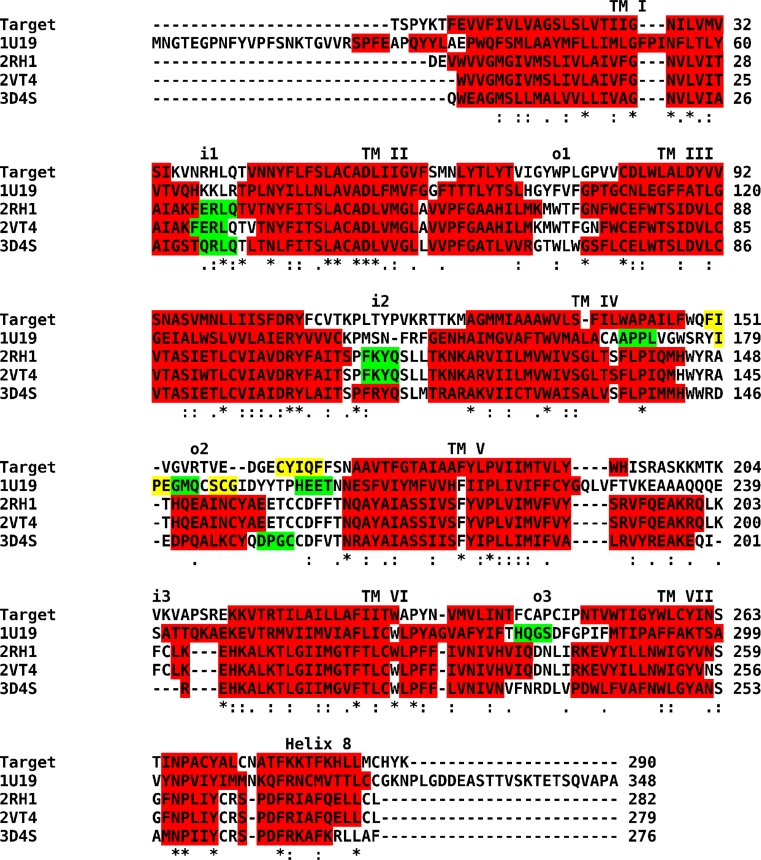
Alignments of templates to target structure. Alignment of templates for homology modeling labeled by their PDB entry code to the target sequence of the human M_2_ muscarinic acetylcholine receptor. *Stars* denote conserved and dots consensual residues. Colors denote secondary structure: *red*—helix; *white*—coil; *yellow*—strand; *green*—turn. Secondary structure of the target was predicted by PsiPred (http://bioinf.cs.ucl.ac.uk/psipred/) and secondary structures of templates were taken from respective crystal structures. For orientation transmembrane (TM) helices, inner (i) and outer (o) loops are indicated

**Fig. 2:**
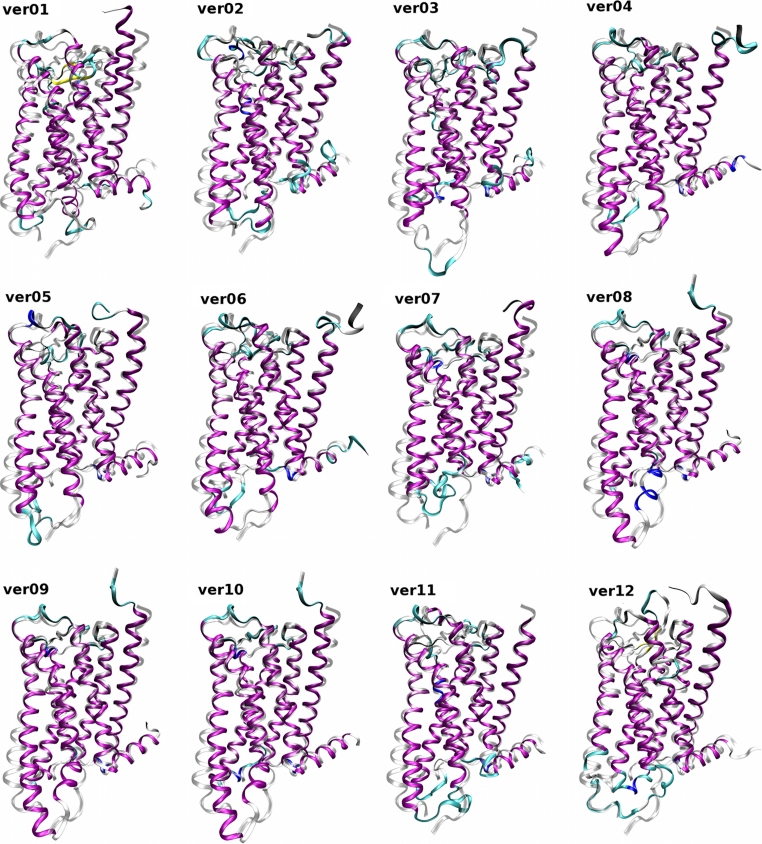
Twelve homology models of the M_2_ receptor superposed on the 3UON crystal structure. Homology models (*color*) of the M_2_ receptor based on the templates listed in Table 1 are superposed using MUSTANG [27] implemented in YASARA on the crystal structure 3UON (*gray*). Orientation: extracellular site up, TM VI and TM VII front. Colors: *purple*—α-helix; *yellow*—β-sheet; *cyan*—turn; *white*—coil. RMSDs of the models to the target structure (3UON) are listed in Table 4

The best templates (available in the beginning of this study) of class A of G-protein-coupled receptors in an inactive conformation [8, 14–16] were aligned to the shortened sequence of the human M_2_ muscarinic acetylcholine receptor (Fig. 1) and several homology models were built (Fig. 2). Three of the models (ver01–ver03) were built on a single template structure using Prime 2.1 [17] (Table 1). Models ver04 and ver05 were built using Modeller [19] or the YASARA [18] implementation of Modeller on 4 template structures (1U19, 2RH1, 3D4S, 2VT4). After publication of the crystallographic structure of the M_3_ muscarinic receptor (4DAJ) additional 6 models were built using this structure as template either as single template (ver07 and ver08) or with additional 5 or 10 structures of GPCRs as templates (ver09 through ver12) using Prime 3.1 or YASARA (Table 1).

**Table 1:** List of models, their templates and the modeling programs that were used

Model	Procedure	Template (s) [PDB codes]
ver01	Prime	1U19
ver02	Prime	2RH1
ver03	Prime	2VT4
ver04	YASARA	1U19, 2RH1, 2VT4, 3D4S
ver05	Modeller	1U19, 2RH1, 2VT4, 3D4S
ver06	YASARA	Hybrid model of ver07 and ver08
ver07	Prime	4DAJ
ver08	YASARA	4DAJ
ver09	YASARA	1U19, 2RH1, 2VT4, 3D4S, 4DAJ
ver10	YASARA	1U19, 2RH1, 2VT4, 3D4S, 3ODU, 3PBL, 3RFM, 3RZE, 3V2Y, 4DAJ, 4DJH
ver11	Prime	1U19, 2RH1, 2VT4, 3D4S, 4DAJ
ver12	Prime	1U19, 2RH1, 2VT4, 3D4S, 3ODU, 3PBL, 3RFM, 3RZE, 3V2Y, 4DAJ, 4DJH

Templates used for first six models (ver01–ver06) share from 24 to 31 % of sequence homology and from 74 to 91 % of secondary structure homology, have 2.8 Å or better resolution. Templates for the last six models (ver07–ver12) have up to 71 % sequence homology and up to 97 % secondary structure homology with the target sequence. Quality checks of the templates are summarized in the Table 2.

**Table 2:** Quality checks of templates and target structure

	Homology	Homology	Resolution	Prime	YASARA
	1D [%]	2D [%]	[Å]	G-factor (+)	Z-score (+)
Templates					
1U19	24	74	2.2	−7.935	−0.733
2RH1	31	89	2.4	−7.313	0.589
2VT4	31	91	2.7	−7.154	0.413
3D4S	31	86	2.8	−7.436	0.788
3ODU	25	73	2.5	−7.808	0.236
3PBL	34	85	2.9	−7.299	0.044
3RFM	29	84	3.6	−7.246	0.086
3RZE	39	89	3.1	−8.043	−0.472
3V2Y	29	73	2.8	−7.365	−0.262
4DAJ	71	97	3.4	−7.778	−0.156
4DJH	31	78	2.9	−7.309	0.118
Target					
3UON	100	100	3.0	−7.585	0.052

Qualities of the templates per cent of sequence (1D) and secondary structure (2D) homology of the templates to the target structure (3UON), resolution of the crystalographic structures in Å, Prime geometry factor (G-factor), and YASARA quality check score (Z-score) are shown. Except for resolution higher is better

### Evaluation of basic models

None of the models contained obvious errors (cis-prolines, side-chain clashes), and according to the Ramachandran plots contained no more than 4 residues in the disallowed region, none of which was in a part of the receptor that is deemed important for binding or activation. All receptor models were stable according to simulation of short 50-ns molecular dynamics. The receptors equilibrated within 10–20 ns from the initial conformation to a conformation with lower energy within 3.5–4.5 Å RMSD of protein heavy atoms and remained as such for the rest of the simulation. A helical bundle was minimally affected by molecular dynamics but the second extracellular loop underwent major rearrangement flipping out of the receptor. Also the second extracellular loop of the target structure flips out during molecular dynamics simulation.

The superposition of homology models on the crystal structure of the M_2_ muscarinic receptor (Fig. 2) shows that these models are correct in the bundle of transmembrane segments, except for the tilt of TM V (plus TM I and TM IV of model ver01). Despite exhaustive loop sampling and refinement, the most obvious divergence from the crystal structure is in the flanking N- and C-termini and the long second extracellular loop (o2) with a marked imprint of the secondary structure of the templates (β-sheet of rhodopsin, α-helix of β-adrenergic receptors). Individual amino acids have correct orientation within the orthosteric binding site and almost all (>98 %) TMs. RMSDs of models ver01 and ver12 differ most from the crystallographic structure of the M_2_ receptor, while models ver07–ver10 differ least (Table 3). This correlation applies to the whole models and structurally aligned residues, and is most eminent for the orthosteric binding site. Disulfide bonds of Cys96–Cys176 were present and the orientation of key amino acids was correct (Ser76, Trp99, Asp103, Tyr104, Thr187, Thr190, Tyr403, Asn404, Tyr426 and Tyr430 at the orthosteric site; Tyr83, Thr84, Asn410, Thr411, Trp422 and Thr423 at the opening of orthosteric site to the extracellular space; Asp69, Ser433 and Asp436 at the activation site; and Asp120, Arg121, Tyr122 and Glu382 at the signal transduction site).

**Table 3:** RMSDs and quality checks of homology models

	RMSD (Å)	Prime G-factor (+)	Prime energy (−)	YASARA Z-score (+)	YASARA energy (−)	Modeller DOPE-score (−)
RMSDs and quality checks of whole models						
ver01	5.611	−8.161	−10,858	−1.602	−101,125	−40,964
ver02	4.366	−7.599	−11,284	−0.487	−115,209	−43,018
ver03	3.799	−7.823	−9,055	−0.836	−109,204	−41,671
ver04	3.538	−7.355	−8,913	0.284	−121,882	−42,550
ver05	3.861	−7.936	−8,252	−1.580	−100,605	−40,929
ver06	3.504	−7.574	−10,213	−0.397	−115,606	−41,124
ver07	2.985	−8.219	−10,842	−0.521	−111,144	−41,559
ver08	2.824	−6.558	−10,494	0.285	−124,144	−42,602
ver09	2.948	−7.425	−10,572	0.193	−124,519	−42,994
ver10	2.958	−7.362	−10,515	0.236	−124,578	−42,883
ver11	3.141	−7.952	−9,693	−1.212	−100,836	−38,448
ver12	5.581	−8.057	−10,094	−1.792	−94,563	−38,189
R	1.00	−0.55	0.06	−0.74	0.71	0.45
*P* value		0.466	1.00	0.058	0.083	0.756
RMSDs and quality checks of orthosteric binding site						
ver01	2.998	−9.323	−733	−0.771	−5,438	−5.99
ver02	2.277	−9.357	−669	−0.132	−6,294	−6.32
ver03	1.803	−9.561	−657	−0.090	−6,372	−6.36
ver04	1.314	−10.02	−506	−0.304	−5,426	−6.52
ver05	1.305	−9.391	−439	−0.458	−5,433	−6.49
ver06	1.318	−9.142	−492	−0.584	−5,700	−6.53
ver07	1.036	−8.249	−528	−0.034	−6,261	−6.44
ver08	0.785	−8.883	−547	0.005	−6,706	−6.73
ver09	1.585	−7.954	−533	0.086	−6,781	−6.79
ver10	1.504	−8.561	−529	0.182	−6,883	−6.81
ver11	1.942	−10.67	−484	−0.980	−5,009	−6.37
ver12	2.602	−10.00	−505	−1.065	−4,588	−6.18
R	1.00	−0.36	−0.32	−0.50	0.27	0.68
*P* value		1.00	1.00	0.666	1.00	0.169

RMSD of homology models to target structure (3UON) is in Å and the results of the quality checks built into the modeling programs are in arbitrary units (G-factor, Z-score, DOPE-score) or in kcal/mol (Prime Energy, YASARA Energy). Prime energy, YASARA energy and DOPE-score—more negative is better (−); G-factor and Z-score—more positive is better (+). R, correlation coefficient of the quality test values to the RMSD values; *P* value, *P* values from Sperman correlation analysis adjusted by Holm’s method

Analysis of major interhelical interactions is summarized in Table 4. In muscarinic receptors the interaction between TM II and TM IV is mediated by hydrogen bonds between Ser64 of TM II and Asn113 and Trp148 in TM IV. This interaction is present in models ver01–ver03, ver07 and ver08, is partial in models ver04–ver06 and absent in models ver09–ver12. Interaction between TM II and TM VII is mediated by hydrogen bonds between Asp69 of TM II and Ser433 and Asn436 of TM VII. This interaction is absent only in model ver12, is partial in models ver04 and ver11. In models ver01, ver06, ver07 and ver10 Asp69 binds to Tyr440 instead of Ser433 or Asn436. A unique interaction between the TM III and o2 loops of muscarinic receptors that affects affinity of orthosteric ligands is mediated by hydrogen bonds between Asp97 at the edge of the TM III and Gln163 and Arg169 of the o2 loop. This interaction is present at models ver07–ver09 and partially at model ver03. At model ver06 Asp97 makes hydrogen bond to Gln179 with substantially altered conformation of the o2 loop. Interaction between TM III and TM IV is mediated by hydrogen bonds between Asn108 of TM III and Ser151 and Trp155 of TM IV. This interaction is present only in model ver07 and partially in models ver03, ver05, ver08, ver11 and ver12. Interaction between TM III and TM VI that keeps the receptor in an inactive conformation is present in models ver03, ver04, ver06–ver08. It should be noted, however, that this interaction is missing in the target structure 3UON. Based on the evaluation of intramolecular interactions none of the models is perfect, however, models ver07 and ver08 seem to be the best ones. Indeed model ver08 has the lowest RMSD to target structure among the 12 models (Table 3).

**Table 4:** Analysis of homology models for major intramolecular interactions stabilizing muscarinic receptors

	ver01	ver02	ver03	ver04	ver05	ver06	ver07	ver08	ver09	ver10	ver11	ver12
TM II–TM IV												
Ser64–Asn113	Y	Y	Y	N	Y	N	Y	Y	N	N	N	N
Ser64–Trp148	Y	Y	Y	Y	N	Y	Y	Y	N	N	N	N
TM II–TM VII												
Asp69–Ser433	Tyr440	Y	Y	N	Y	Tyr440	Y	Y	N	N	N	N
Asp69–Asn436	Y	Y	Y	Y	Y	Y	Tyr440	Y	Y	Tyr440	Y	N
TM III–o2												
Asp97–Gln163	N	N	N	N	N	Y	Y	Y	Y	N	N	N
Asp97–Arg169	N	N	Y	N	N	Gln179	Y	Y	Y	N	N	N
TM III–TM IV												
Asn108–Ser151	Thr190	N	Y	N	Y	N	Y	Y	N	N	Y	Y
Asn108–Trp155	N	N	N	N	N	N	Y	N	N	N	N	N
TM III–TM VI												
Arg121–Glu382	Ser118	Asp120	Y	Y	Asp120	Y	Y	Y	N	N	N	Asp120

Existence of the hydrogen bond between amino acid pair shown in row at model shown in column is indicated as yes (Y), no (N) or interaction with alternative amino acid

The models differ substantially: the calculated RMSDs varied from 2.8 to 5.6 Å for whole models and from 0.8 to 3.0 Å for an orthosteric binding site (Table 3). The models differ substantially in structurally aligned residues (residues sharing the same secondary structure): RMSDs varied from 1.1 to 2.0 Å (Table 5). Not surprisingly, the variations in the RMSDs show major impact of the template on the model. The structurally most distant template (rhodopsin, 1U19) results in the model with the highest RMSD (ver01, 5.6 Å) while the structurally closest template (M_3_ muscarinic receptor, PDB:4DAJ) results in the lowest RMSD (ver08, 2.8 Å). Quality of the models was assessed by internal quality checks scoring model geometry and by calculation of model energies implemented in the modeling programs Prime, YASARA and Modeller (Table 4). None of the quality checks correlated with model RMSD to target structure. The orthosteric binding site for muscarinic agonists and antagonists is located approximately in the middle of the membrane lipid bilayer, among the receptor transmembrane helices. There is a higher chance of a more accurate model as it is approximately in the center of the templates. However, like the quality checks of whole models, the scores of individual built-in quality checks did not correlate with RMSD of the orthosteric binding site of the model to target structure.

**Table 5:** RMSDs of individual homology models and the best docking poses of QNB versus the target structure

	Whole models	Structurally aligned residues	Binding site free	Binding site docked	QNB
ver01	5.611	1.957 (183)	2.998	2.777	4.772
ver02	4.366	1.668 (238)	2.277	2.036	4.879
ver03	3.799	1.684 (238)	1.803	2.024	3.043
ver04	3.538	1.471 (237)	1.314	0.983	1.890
ver05	3.861	1.462 (238)	1.305	0.918	1.549
ver06	3.504	1.455 (238)	1.318	0.978	1.135
ver07	2.985	1.354 (244)	1.036	0.446	0.481
ver08	2.824	1.080 (254)	0.785	0.402	0.523
ver09	2.948	1.220 (250)	1.585	0.856	1.269
ver10	2.958	1.208 (253)	1.597	1.593	1.197
ver11	3.141	1.451 (229)	1.942	1.268	3.502
ver12	5.581	1.579 (199)	2.602	1.992	5.293

The values are the RMSDs in Å. The value in parenthesis represents the number of structurally aligned residues by MUSTANG. The binding site is Ser76, Trp99, Asp103, Tyr104, Thr187, Thr190, Tyr 403, Asn404, Tyr 426, and Tyr430 (M_2_ sequence)

The quality of the orthosteric binding site was further probed by docking the muscarinic antagonist *N*-methylscopolamine (NMS), using either Glide [20] or Autodock [21]. Docking NMS produced reasonable poses with hydrogen bonding to Asn404 (except ver01 and ver02, data not shown). NMS docking to ver04 produced better hydrogen bonding of NMS to Asp103, while NMS docking to model ver05 produced better hydrogen bonding to Asn404.

### Re-docking of QNB to 3UON

**Fig. 3:**
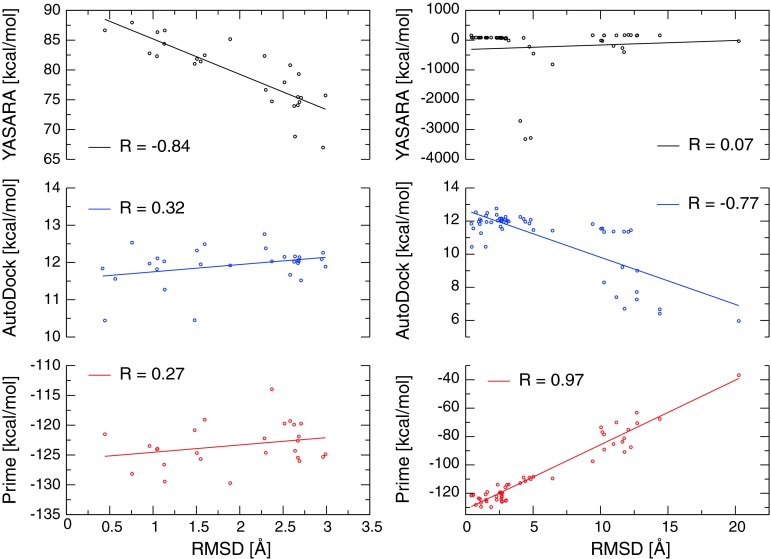
Correlation of calculated binding energies and RMSD of redocked QNB to 3UON. Calculated YASARA binding energies (*top*), AutoDock energies (*middle*) and Prime MMGB/SA energies (*bottom*) of poses from QNB re-docking to 3UON are plotted against their RMSDs. Detail with poses of RMSD of ligand smaller than 3 Å is on left and all poses are on right. Correlation coefficients are indicated in the graphs

Because quality checks fail in evaluation and ranking of the models some other approach for model evaluation would be helpful. Fortunately, the crystallographic structure of M_2_ muscarinic receptor (3UON) contains the antagonist quinuclidinyl bezilate (QNB). We re-docked QNB to the original structure using either Prime or Autodock. Resulting poses from re-docking by both procedures were evaluated by Prime MMGB/SA and YASARA binding energy function and these estimates were compared with RMSD of the resulting pose. There is good correlation between Prime relative binding energy estimation and pose RMSD but YASARA binding energy function lacks this correlation (Fig. 3 right). However, if only poses with RMSD lower than 3 Å are taken situation is completely opposite. YASARA binding energy function correlates well with pose RMSD but Prime binding energy estimation does not (Fig. 3 left). This suggests that a pose with a best estimate according YASARA chosen among poses well scoring according Prime is likely to be one of the best poses. If this approach works for other ligands than relative binding affinities of correct poses of docked ligands may be compared. It has been shown that MMGB/SA predicts the correct relative binding energies of ligands for known structures [22, 23], so if the MMGB/SA based relative binding energies for a given model are correct, then the model itself is correct, at least in regard to the binding site.

### Induced fit docking and binding energy estimation

It is obvious that structurally different ligands should induce different conformations of the binding site, and that the conformation induced by a given ligand accommodates this ligand best. As a preliminary test of the concept, induced fit docking of the antagonists NMS to homology models produced better results than mere docking using Glide or Autodock. Therefore, induced fit docking was implemented using either Schrödinger’s “Induced Fit Docking Workflow” or according to the procedure of Naburs et al. [24] of four non-selective antagonists of muscarinic acetylcholine receptors: QNB, *N*-methylquinuclidinyl benzilate (NMQNB), NMS, and Atropine (Atrop) with known affinities of 74, 120, 260, and 490 pM, respectively, to all homology models. The resulting top poses from both docking procedures were pooled and inspected visually. All poses bound to the receptor with at least one hydrogen bond. No steric clashes or other obvious errors were detected.

**Fig. 4:**
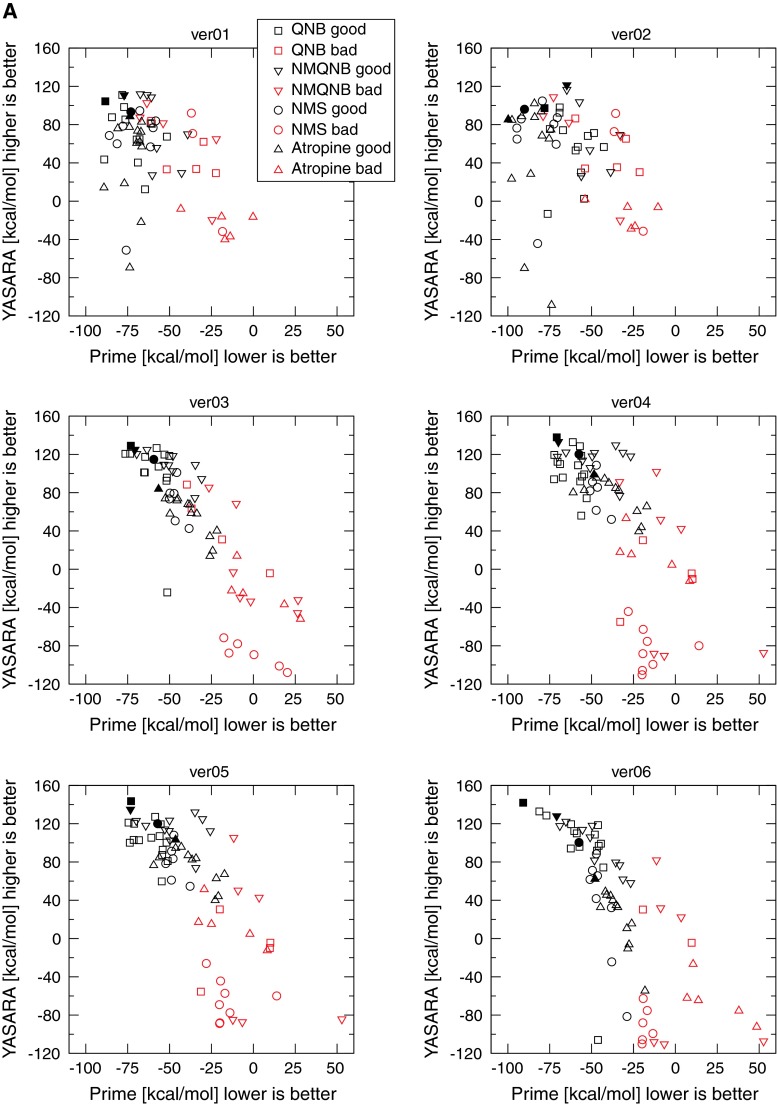

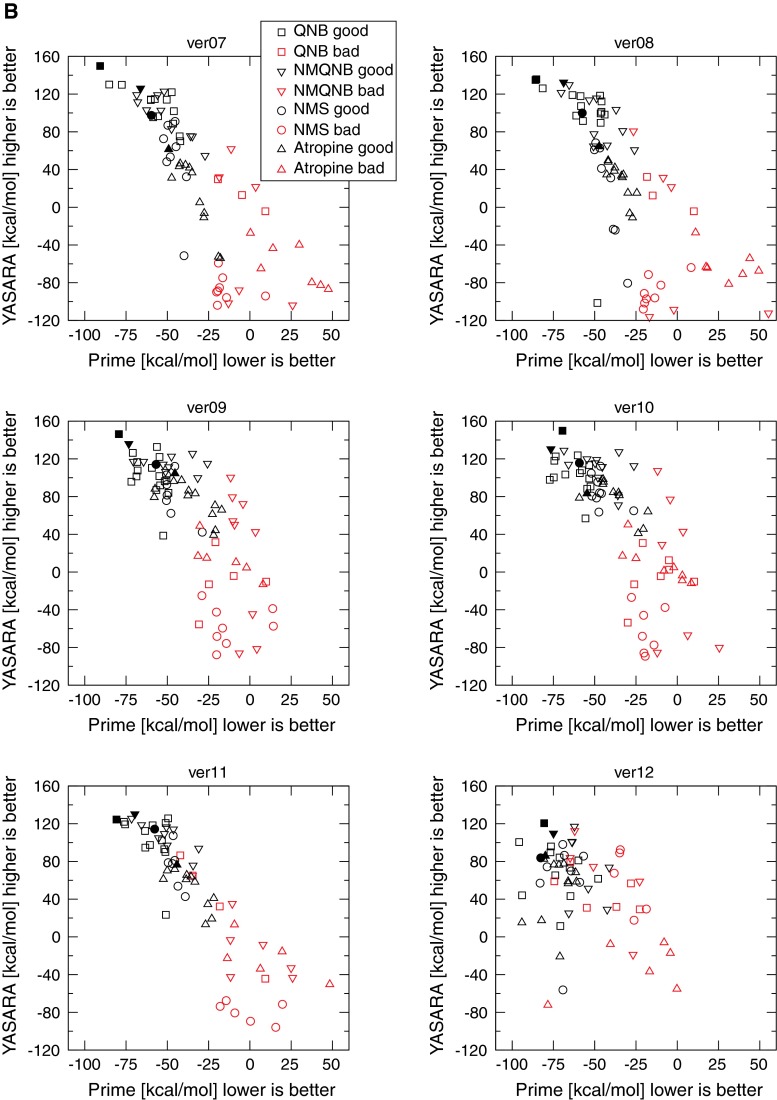
Calculated binding energies of muscarinic antagonists docked to homology models. Calculated binding energies of the antagonists QNB (*squares*), NMQNB (*downward triangles*), NMS (*circles*) and atropine (*upward triangles*) in YASARA (*y-axis*) are plotted against the binding energies calculated in Prime MMGB/SA (*x-axis*). Good poses are *black*, and bad poses are *red*. *Closed symbols* denote the best poses

Poses were labeled either “bad” or “good”. According to Schulman’s model of the muscarinic pharmacophore [25] two interactions are essential for muscarinic orthosteric ligands. The nitrogen head-group interacts with an aspartic acid residue while a region of negative electrostatic potential interacts with a positive receptor residue and forms a hydrogen bond with it. It was confirmed experimentally that the nitrogen group of muscarinic orthosteric ligands interacts with an aspartate in TM III (D3.32) [26–29] and part of the ligand with negative electrostatic potential of antagonists interacts with an asparagine in TM VI (N6.52) [30, 31]. Formation of hydrogen bond with an asparagine in TM VI (N6.52) was confirmed by crystallography [5, 6]. Although contribution of individual amino acids to binding varies among individual ligands [26, 31] the orientation of all ligands in the respect to these two key amino acids is the same for all ligands [32]. These two major interactions define the orthosteric binding site and ligand orientation. Poses that have the ligand fully or partially outside the expected binding site, or poses where the ligand is in the expected binding site but in the wrong orientation (e.g. the nitrogen group oriented towards TM VI, region of negative electrostatic potential interacting with tyrosine in TM III (Y3.33), etc.) were labeled as bad. The binding energies were calculated for all top poses using both Schrödinger’s Prime MMGB/SA and the YASARA binding energy function, and are plotted in Fig. 4. The poses with highest YASARA energy among top-scoring poses in Prime MMGB/SA were labeled “the best”. The worst binding energy calculation results were obtained for single template model ver01 based on rhodopsin structure and multiple template model ver12 (Fig. 4A upper left, B lower right). The estimates of the binding energies do not discriminate between good and bad. Their calculated binding energies are the same or similar, and the “best” poses do not follow the correct order of the relative binding energies. Interestingly, the same applies for the single template model ver02 (Fig. 2 upper right), which is based on the human β_2_-adrenergic receptor structure (2RH1), though the β_2_-adrenergic receptor is structurally closer to the muscarinic receptor than to rhodopsin. Better results were obtained for a single template model ver03 based on the turkey β_1_-adrenergic receptor structure (2VT4). In this model good and bad poses were separated in the binding energy estimates. However, the estimated relative binding energies of the best poses did not correspond to the experimental data. The better scores of model ver03 (in comparison to ver02) may be due to receptor stabilizing interactions present in the crystal structure of the template (namely R 3.50–E 6.30) that organize a bundle of helices in the correct way. Slightly better scores than those for model ver03 were obtained for multi-template models ver04 and ver05 and ver09–ver11. In comparison with model ver03, they provided better separation of good and bad poses on the basis of an estimation of the binding energies, and a slightly better order of the relative binding energies of the best poses. The better scores of models ver04 and ver05 in comparison with the scores for models ver01 through ver03 may be attributed to multiple templates. The multiple template-based model ver06 (Fig. 4A lower right) and single template models ver07 and ver08 (Fig. 4B middle) based on the closest structure of M_3_ muscarinic receptor are the best according to the estimated binding energies. In this model, good and bad poses are well separated by the binding energy estimates, and the estimated relative binding energies of the best poses are in good agreement with the experimental data.

Importantly, the worst-scoring models according to binding energy estimation analysis (ver01 and ver12) show the largest deviations from the crystallographic structure, while the best-scoring models (ver07–ver10) show the smallest deviations. The estimate of the binding energies thus can roughly distinguish bad models from relatively good ones, is beneficial in excluding bad models but is not sufficient for the identification of the best model.

The binding energy calculations of Prime and YASARA ignore entropic components, and thus are not suitable for absolute energy estimations. Indeed, the absolute binding energy values of the best poses in the range from 140 to 60 kcal/mol are overestimated by 5–10 times (Fig. 4). The binding energy values for QNB, NMQNB, NMS and atropine derived from the experimental data are 13.8, 13.5, 13.1, and 12.7 kcal/mol, respectively. Autodock adds an entropic component to mechanistic terms of binding energy and estimates the binding energies more accurately: 12.9–12.1, 12.4–11.5, 11.6–10.8 and 11.1–10.2 kcal/mol for top 10 poses of QNB, NMQNB, NMS and atropine, respectively. However, AutoDock does not discriminate between correct and wrong poses (the estimates of binding energies are the same for correct and wrong poses) and relative affinities are overlapping and thus cannot be taken for model evaluation. It seems that the contribution of the entropic component “masks” differences in the mechanistic component that is important for correct estimation of relative binding energies and subsequently model evaluation.

**Fig. 5:**
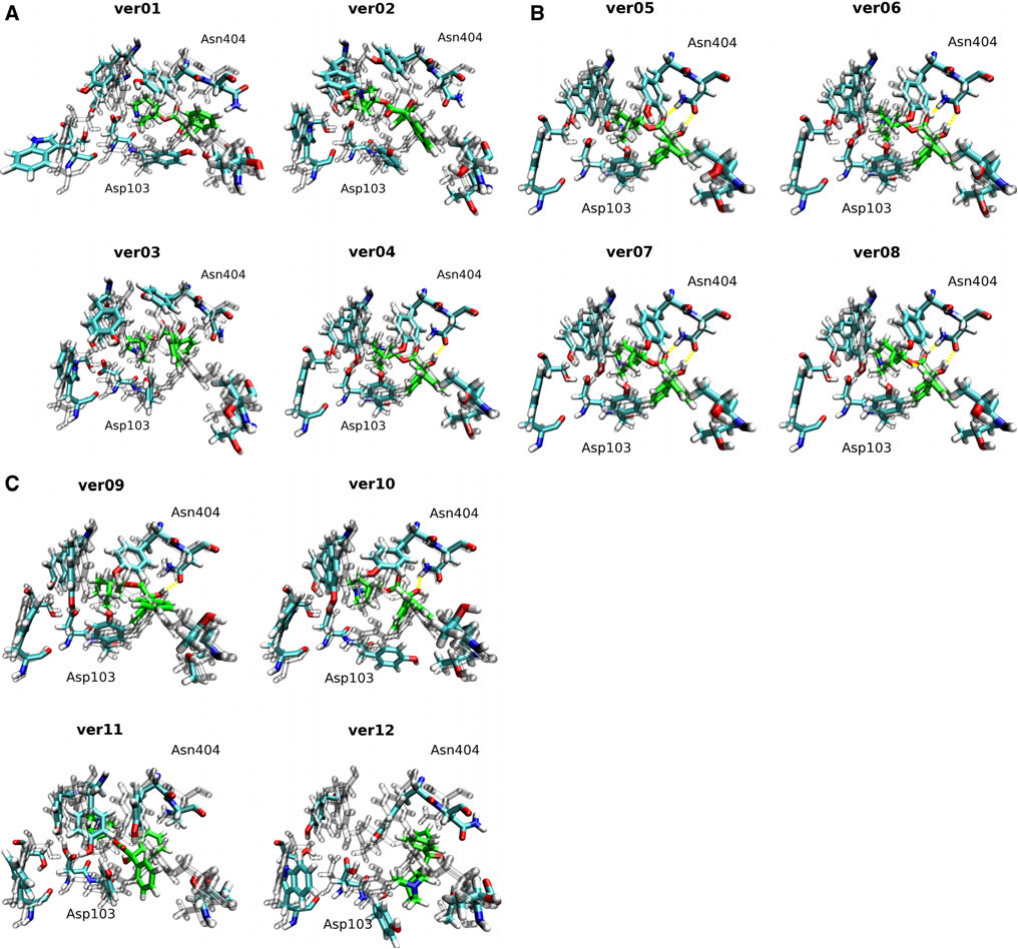
The best QNB docking poses of 12 homology models superposed on the 3UON crystal structure. View from the extracellular site, TM II down, TM VI and TM VII up, of the best docking poses, according to the binding energy estimates (Fig. 4), of QNB (*green carbons*) and residues of the orthosteric binding site (*color*) superposed on the crystal structure of 3UON (*gray*). Colors: *Cyan*—carbon; *red*—oxygen; *blue*—nitrogen; *white*—hydrogen; *yellow*—hydrogen bonds. The residue labels correspond to the M_2_ sequence. The calculated RMSDs of QNB and residues of the orthosteric binding site of the models to the target structure (3UON) are shown in Table 5

When compared to ligand-free models induced fit docking of QNB (Fig. 5) itself further lowered the RMSDs of the orthosteric site of the models, with the exception of model ver03 (Table 5). The RMSDs of the docked QNB to the target structure 3UON are highest (over 5 Å) in model ver12, relatively high (3.0–4.9 Å) in models ver01–ver03 and ver11, markedly lower (1.2–1.9 Å) in models ver04–ver06, ver09 and ver10 and lowest (about 0.5 Å) in models ver07 and ver08 (Table 5, last column). The models with overall lowest RMSD to target structure (ver07 and ver08) have also the ligand with the lowest RMSD to the target structure.

Simulation of molecular dynamics of homology models with bound QNB (in the best pose) shows that models are stable and as expected more rigid than models without ligand. The ligand–receptor complex equilibrates more slowly (about 20 ns) and the equilibrium conformation is closer to the initial structure (RMSDs of protein heavy atoms <3.0 Å) than models of empty receptors. Although the model improves during simulation (total energy of the system decreases) there is no decrease in RMSD to the 3UON structure.

## Summary

The data clearly show that: (1) Accuracy of homology models is determined by the template. The best model was based on single template with the highest homology to the target structure. Including additional templates worsened the results. (2) The influence of the template on the resulting model is most marked in parts that differ in the secondary structure, and these differences cannot be overcome by computing. (3) The model quality checks built into the programs are only approximate, and cannot be used for choosing the best model. (4) The only way to select an overall good model is visual inspection for known structural features, intramolecular interactions, hydrogen bond networks, etc.

The analysis of the estimated binding energies may help in judging the quality of the model biding site by excluding bad models, albeit with some precautions. First precaution is that this analysis applies only to the ligand binding site and its immediate vicinity. The second caveat of this approach is the conformational change effected by induced fit docking. While it is obvious that an induced fit of the binding site is essential to accommodate structurally different ligands, excessively large conformational changes in the receptor structure may flaw the binding energy calculations by the contribution of the conformational change to the binding energy. This contribution is certainly large, but its exact size is unknown. Thus only structurally similar ligands should be compared, and conformation changes induced by ligand docking should be kept to a minimum.

The recently published crystallographic structures of the M_2_ and M_3_ receptor are practically identical in the secondary structure [5, 6]. Data suggest that modeling the remaining three muscarinic receptor subtypes or mutant receptors based on these two structures will very likely result in good models and other templates should not be included in modeling procedure. However, simple homology modeling is with high probability unsuitable for modeling of ligand binding that induce large conformational change (e.g. muscarinic allosteic modulators; for a review see [2]). As noted above success or failure of simple homology modeling is determined by the suitability of the template(s). A suitable template for this task (e.g. crystal structure of muscarinic receptor with bound allosteric ligand) has not been published so far. Furthermore, large conformational changes impede utilization of binding energy calculations in evaluation of potential models.

## Methods

### Preparation of templates

The high-resolution structures of closely related GPCR in the inactive conformation available in the beginning of this study were downloaded from the RCSB Protein Data Bank. Eleven homology modeling templates were chosen due to the high resolution and high homology with the M_2_ muscarinic receptor: bovine rhodopsin (PDB:1U19) [8], human β_2_-adrenergic receptor T4-lysozyme chimera (PDB:2RH1 and PDB:3D4S) [14, 15], turkey β_1_-adrenergic receptor with stabilizing mutations (PDB:2VT4) [16], CXCR4 chemokine receptor (PDB:3ODU) [33], human dopamine D_3_ receptor (PDB:3PBL) [34], adenosine A_2A_ receptor (PDB:3RFM) [35], human histamine H_1_ receptor (PDB:3RZE) [36], sphingosine 1-phosphate receptor 1 (PDB:3V2Y) [37], M_3_ muscarinic receptor (PDB:4DAJ) [6], and human κ-opioid receptor (PDB:4DJH) [38]. In case of multimers single chains with the best resolution were chosen and the rest (water, lipids, ions, fusion protein, etc.) were deleted. Templates were processed with the Schrodinger Suite protein preparation wizard. Then the templates were inspected for major intramolecular interactions that stabilize the receptor structure [10]: 2.45–4.50, 2.50–7.49 and 3.50–6.30 (numbering according to Ballesteros and Weinstein [39]).

### Building the models

A human M_2_ muscarinic receptor with truncated N- and C-termini and an i3 loop was modeled. For single template models built by Prime or the multiple template model built by Modeller the modeled sequence was manually aligned to the templates according to so-called pin-points [40] (Fig. 1). For multiple template models built by Prime or YASARA the modeled sequence was aligned by modeling programs.

Several models were built using Prime [17], Modeller [19] and/or YASARA [18] (Table 1). The initial crude models were checked for major intramolecular interactions that stabilize the receptor structure, and were subjected to the basic quality checks built into the modeling programs. For the initial crude models that scored the best in the quality checks, alternative N and C termini and intra- and extracellular loops were modeled. The best models were then combined to final models that were refined and energy minimized (Table 1).

### Evaluation and comparison of the models

The final models were examined for possible errors, for disallowed conformation of residues using the Ramachandran plot, and the presence of conserved receptor stabilizing interactions was checked again. All final models were cross-evaluated by the built-in quality check procedures of all three modeling programs: Prime G-factor and total energy, YASARA Z-score and Potential energy and Modeller DOPE-score. The models were either evaluated as a whole, or only the orthosteric binding site was evaluated. In the case of the orthosteric binding site, scores for residues Ser76 (2.57), Trp99 (3.46), Asp103 (3.32), Tyr104 (3.33), Thr187 (5.43), Thr190 (5.46), Tyr403 (6.51), Asn404 (6.52), Tyr426 (7.39), and Tyr430 (7.43) were calculated (the M_2_ sequence, in parenthesis is numbering according Ballesteros and Weinstein [39]).

The stability of the final models was checked by 50-ns molecular dynamics simulation in an explicit DPPC membrane/water/0.15 M NaCl environment, using Desmond [41] (version 2.4) and newest (2005) version of OPLS-AA force field. The models were inserted into a DPPC bilayer, the charges were neutralized, the simulation box with periodic boundaries was filled with water, and the concentration of Na^+^ and Cl^−^ ions increased to 0.15 M. The models were first relaxed with the Desmond procedure for membrane proteins, which prevents water entering the membrane, and then 50 ns of molecular dynamics were simulated. The simulations were performed in NPT ensemble. A temperature of 325 K and a pressure of 1.01325 bar were kept constant by coupling to a Berendsen thermostat and barostat. Integration step was 2.0 fs. The cutoff radius for Coulombic interactions was 9.0 Å. Long-range electrostatic interactions were calculated using the smooth particle mesh Ewald method.

For comparison the models were structurally aligned by MUSTANG [42], and then the RMSDs of whole models and structurally common parts were calculated. Alternatively, the models were aligned according their orthosteric binding sites, and the RMSDs of the residues (see the list above) in the orthosteric binding site were calculated.

### Docking of antagonists

Three-dimensional structures of antagonists of the muscarinic receptors QNB, *N*-methyl-quinuclidinyl benzilate (NMQNB), *N*-methyl-scopolamine (NMS) and atropine (Atrop) were downloaded from Pubchem database, pre-processed by Schrödinger LigPrep or YASARA, and docked to the orthosteric binding site (residues Ser76, Trp99, Asp103, Tyr104, Thr187, Thr190, Tyr403, Asn404, Tyr426, Tyr430, M_2_ sequence) of the homology models, using either Schrödiger Glide [20, 43] or YASARA implementation of Autodock [44]. Glide grid was set to residues of orthosteric binding site or in case of QNB-redocking to the QNB of crystal structure and size of binding site was set to “Auto” and size of the ligand was set to “similar size”. Serine, tyrosine and threonine hydroxyl groups were alloved to rotate. Glide docking was set to extra precision (XP) and constrained to poses having H-bond between ligand and residue Asp103 or Asn404. The best 10 poses according to the Glide XP score or the best poses within a 10 kcal/mol (Glide energy function) range were further evaluated. In the YASARA implementation of AutoDock 500 runs were made for each ligand and model combination, with the following parameters: rmsdmin for clusters was set to 2.0 Å, the force field was AMBER03, and AutoDock method was Lamarckian Genetic Algorithm (LGA) [21]. The best 10 complexes according to the AutoDock score or within 1 kcal/mol (AutoDock energy function) were taken for further analysis.

### Induced fit docking of antagonists

QNB, NMQNB, NMS and atropine were docked to homology models using either the Schrödinger Induced Fit Docking procedure or the protocol according to Nabuurs et al. [24], implemented to YASARA/Autodock. In the Schrödinger Induced Fit Docking procedure the same residues as in simple docking were chosen to define the orthosteric binding site, docking was constrained to the H-bond between ligand and residue Asn404, initial docking was with standard precision (SP), Prime optimization was a double pass for residues within 5 Å distance, and the final docking was with XP. Alternatively, ligands were docked to models using Autodock LGA (version 4.2) with residues in the binding site marked as flexible. The top 10 complexes or complexes within 1 kcal/mol range were further refined by the steepest descent energy minimization in vacuo Yamber 2 force field [45], followed by simulated annealing. Refined complexes were re-docked to rigid protein using the AutoDock Local Search method.

### Estimation of relative binding energies

The ligand/receptor binding energies were calculated either using Prime implementation of MM/GBSA (version 1.41) or YASARA. In Prime MM/GBSA, the protein was kept rigid in implicit membrane, and the strain energies were included in the calculations. The solvent model was vbgs2.0. In Prime the binding energy is calculated as the difference between MM/GBSA energy of the complex and the sum of MM/GBSA energies of the unliganded receptor and the free ligand. Thus more negative energy (difference) means higher affinity. In the YASARA binding energy function, the energy is calculated as the difference between the sum of potential and solvatation energies of the separated compounds and the sum of potential and solvatation energies of the complex in the YAMBER3 force field. Thus more positive energy (difference) means higher affinity.

### Experimental measurement of binding affinities

Affinities of QNB, NMQNB, NMS and atropine were determined in saturation binding experiments of membranes from CHO cells stably expressing M_2_ receptors by tritiated ligands (Amersham). CHO cells were harvested by mild trypsinization, washed in phosphate buffered saline (pH = 7.4) by centrifugation 3 min at 250×*g*, cooled on ice, homogenized by two 30 s strokes in thurrax homogenizer in ice cold homogenization medium (100 mM NaCl, 10 mM MgCl_2_, 10 mM EDTA, 20 mM Na-HEPES buffer pH = 7.4), centrifuged 5 min at 1,000×*g*, supernatant was taken and centrifuged for 30 min at 30,000×*g*, pellets were resuspended in incubation medium (100 mM NaCl, 10 mM MgCl_2_, 20 mM Na-HEPES buffer pH = 7.4), incubated on ice for 30 min, centrifuged for 30 min at 30,000×*g*. Pellets were stored at −80 °C until binding experiment. Saturation binding experiments were carried out on 96-well plates at final volume of 0.8 ml of incubation medium at 25 °C. Non-specific binding was determined in the presence of 10 μM atropine. Incubation lasted 3 h. Incubation was terminated by fast filtration (lasting 6 s) through Whatman filtration GF/C plates on Brandell cell harvester. After drying 50 μl of liquid scintillating cocktail (Rotiszint) was added to each sample on filtration plate. Retained radioactivity was measured on Wallac Microbeta counter.

## Abbreviations

CHO: Chinese hamster ovary
NMS: *N*-methylscopolamine
NMQNB: *N*-methylquinuclidinyl benzilate
QNB: Quinuclidinyl benzilate
